# The American Surgical Association

**Published:** 1893-06

**Authors:** 


### THE AMERICAN SURGICAL ASSOCIATION.

THE American Surgical Association, whose annual meeting occurs in Buffalo, May 30, 31, and June 1, 1893, was organized in 1879 at the suggestion of the late Dr. S. D. Gross, who had long cherished it as one of the objects of his ambition to establish an association which should bear a national name, and embody in one harmonious whole the leading surgical talent, experience, and wisdom of this great country.

He first took into his confidence Drs. Moses Gunn, W. T. Briggs, and W. W. Dawson, who, with him, may be considered as the founders and organizers of the Association.

In May, 1880, on the invitation of the above-mentioned gentlemen, about fifty of the leading surgeons of this country assembled in Philadelphia, formally organized the Association, and elected Dr. Gross as their first president. The next meeting of the Association was held in Richmond, Va., in 1881, while another meeting was held the same year, in September, at Coney Island, at which Dr. Gross was reëlected as president. The third annual meeting was held in Philadelphia in 1882. In his opening address, Dr. Gross disclaimed any intention of injuring or interfering with the surgical section of the American Medical Association, and voiced the hope that the "American Surgical Association might be the altar upon which the surgeons of this country would annually lay their contributions to surgical science, and so show the world that they are earnest and zealous laborers in the interest of human progress and human sufferings."

In 1883, the Association met in Cincinnati, and in 1884, and for eight years thereafter, their annual meetings were held in Washington. Last year they met in Boston, and this year they are to convene in our own city.

Their roll of membership includes almost every name well known in the annals of American surgery, and a membership in their ranks is justly regarded as the highest reward for original and meritorious work in the gift of the American profession.

Through the efforts of Dr. Park, this city was selected as their next meeting place, and it is to be hoped that, inasmuch as their sessions are open to all, the profession of Buffalo and vicinity will turn out in large numbers to meet and greet the men whose names are so familiar to them, and will extend to them a cordial welcome.

We are requested to say that an invitation to attend the scientific sessions of the Association is hereby extended to all, and that they will be held in the Alumni Hall of the University of Buffalo, on the days above mentioned.

The completed programme of the proceedings will be found elsewhere in this number.

